# Intraoperative use of a functional lumen imaging probe during peroral endoscopic myotomy in patients with achalasia: A single-institute experience and systematic review

**DOI:** 10.1371/journal.pone.0234295

**Published:** 2020-06-09

**Authors:** Hyeon Jeong Goong, Su Jin Hong, Shin Hee Kim

**Affiliations:** Department of Internal Medicine, Digestive Disease Center and Research Institute, Soonchunhyang University College of Medicine, Bucheon, Korea; Baylor College of Medicine, UNITED STATES

## Abstract

**Aim:**

The functional lumen imaging probe (FLIP) is a recently developed technique to evaluate the esophagogastric junction (EGJ) distensibility. Unlike timed barium esophagogram (TBE) and high-resolution manometry (HRM), FLIP can be used during peroral endoscopic myotomy (POEM). The aim of this study was to evaluate the association of intraoperative FLIP parameters with clinical outcomes as recorded in a single-center database and to investigate a systematic review of literatures.

**Methods:**

We reviewed consecutive patients diagnosed with achalasia and scheduled for POEM between June 2016 and March 2019 in our tertiary referral hospital. All patients underwent intraoperative FLIP assessment during POEM. The final FLIP measurements were compared between the patients with good and poor clinical response. We comprehensively reviewed studies evaluating whether intraoperative FLIP measurements reflected clinical outcomes.

**Results:**

We evaluated 23 patients with achalasia who underwent intraoperative FLIP before and after POEM. Two exhibited poor clinical responses after 3 months (Eckardt scores = 3). The final distensibility index (DI) did not differ significantly between good and poor responders (5.01 [4.52] vs. 4.91 [3.63–6.20] mm^2^/mmHg at a balloon distension of 50-mL, median [IQR], P = 0.853). The final DI did not differ significantly between post-POEM reflux esophagitis and non-reflux esophagitis groups (6.20 [5.15] vs. 4.23 [1.79] mm^2^/mmHg at a balloon distension of 50-mL, median [IQR], P = 0.075).

**Conclusions:**

A systematic review of both prospective and retrospective studies including our data indicated that the final intraoperative FLIP measurements did not differ significantly between good and poor responders. Further study with more patients is necessary to explore whether FLIP can predict short- and long-term clinical responses.

## Introduction

Achalasia is a rare esophageal motility disorder characterized by impaired relaxation of the lower esophageal sphincter (LES) and the absence of peristalsis. Functional loss of inhibitory neurons of the distal esophagus leads to dysphagia, chest pain, regurgitation, and weight loss. The pathophysiology underlying the symptoms of achalasia is a disturbance in bolus transit through the esophagogastric junction (EGJ). The physiologic components contributing to EGJ bolus flow are peristaltic intraluminal closure pressure, intrabolus pressure, and EGJ opening [1]. As LES relaxation plays an important role in EGJ opening, high resolution manometry (HRM) currently serves as the gold standard in the diagnosis of achalasia and the evaluation of therapeutic outcome. However, several studies have shown that EGJ distensibility also contributes to EGJ emptying [2, 3]. EGJ distensibility means the response of the EGJ to increased intraluminal pressure. Previous studies showed a correlation between EGJ distensibility and symptoms, radiologic findings, and manometric findings in patients with achalasia [2, 3]. In another study, EGJ distensibility supported a diagnosis of achalasia in patients with typical clinical and radiological features of achalasia but without abnormalities on manometry [4].

Functional lumen imaging probe (FLIP) is a newly developed technique that uses high-resolution impedance planimetry during volumetric balloon dilatation to evaluate EGJ distensibility [5]. As obstruction of EGJ outflow is a principal pathophysiology of achalasia, FLIP has been used for both diagnosis and assessment of the therapeutic outcome. Previous studies evaluating FLIP measurements in patients with achalasia revealed that the EGJ distensibility index (DI) was significantly lower in treatment-naïve patients (compared to treated patients), and FLIP parameters improved after treatment [2, 3]. A recent study found that an impaired EGJ distensibility evident on FLIP assessment distinguished achalasia patients with typical symptoms and TBE findings, but manometrically normal LES relaxation, from others [4]. Further, the extents of laparoscopic Heller myotomy (LHM) and peroral endoscopic myotomy (POEM) increased the EGJ-DI in real time [6–8]. Thus, if a FLIP measure associated with symptom improvement and reduced postoperative reflux esophagitis could be defined, EGJ-DI assessment during operation might serve as a valuable form of intraoperative guidance (allowing tailored myotomy). A recent study suggested that the final intraoperative EGJ-DI should be 4.5–8.5 mm^2^/mmHg [9]. but it remains unclear whether the final FLIP parameter predicts the clinical outcome. Also, the optimal FLIP parameters according to different therapeutic procedures, such as LHM, POEM, or PD, has not been evaluated yet.

Given the lack of data on the possible utility of intraoperative FLIP measurement, especially during POEM, we evaluated the association of FLIP parameters assessed during POEM with clinical outcomes. We interrogated a single-center database and systematically reviewed the literature to provide current research on the role of intraoperative FLIP measurement.

## Methods

### Patients

We reviewed consecutive patients diagnosed with achalasia and scheduled for POEM between June 2016 and March 2019 in our tertiary referral hospital. All patients underwent intraoperative FLIP assessment during POEM. The diagnosis of achalasia was confirmed based on the presence of typical symptoms, endoscopy to exclude mechanical obstruction or an inflammatory process, and the results of HRM. The achalasia subtype was determined according to Chicago Classification v3.0 [10]. This study was performed in accordance with the ethical principles of the Declaration of Helsinki and was approved by the institutional review board of Soonchunhyang University Hospital, Bucheon, Korea (SCHBC-2013-02-010-011). Written informed consent was obtained from all patients before their procedures.

### Peroral endoscopic myotomy

Patients underwent POEM after being administered general anesthesia via endotracheal intubation. All procedures were performed by an expert endoscopist who had performed 80 cases of POEM. During the procedures, a commercial CO2 insufflation system (EndoCO2 PRO-600; Miraemedics, Seongnam, Korea) was used to dilate the esophageal lumen. A high-definition endoscope (GIF-Q260J; Olympus, Tokyo, Japan) fitted with a transparent oblique cap (MH-588; Olympus) on its distal end was inserted into the esophagus, followed by the injection of a hypertonic saline, mixed with epinephrine and indigo-carmine solution, into the posterior wall (5 o’clock position) ≥ 12 cm proximal to the EGJ. A hybrid knife (T-type; ERBE, Tübingen, Germany) or hook knife (KD-620 LR; Olympus) was used to perform a mucosotomy on the submucosal bleb via a submucosal tunnel. A myotomy through the submucosal tunnel was then performed with the same knife. In type I and II achalasia, dissection of the inner circular muscle layer extended to at least 2 cm below the EGJ and 6–8 cm above the EGJ. In type III achalasia, we decided the proximal extent of myotomy according to the spastic segment on HRM. The completion of myotomy was confirmed by the reduced scope resistance. After completion of the dissection, the opening of the submucosal tunnel was closed with hemostatic clips (EZ-CLIP, HX-110LR; Olympus) [11].

### Intraoperative impedance planimetry

Intraoperative EGJ distensibility was measured using the EndoFLIP system (Crospon Medical Devices, Galway, Ireland) and FLIP probe (EF-325N; Crospon). The latter contains 18 ringed impedance electrodes spaced at 5 mm intervals within an 8 cm—long polyurethane bag placed on the distal end of the catheter. The probe was prepared as the pressure transducer was zeroed to atmospheric pressure before probe insertion. Prior to the POEM procedure, the FLIP probe was inserted through the gastroscope to the EGJ. The location of the probe was confirmed endoscopically and by the appearance of an “hourglass” configuration on the FLIP monitor. The gastroscope was then removed and the saline filled bag distended from 30-mL, to 40-mL, and 50-mL. Most of the measurements were made using a FLIP bag with 50-mL of distension. The narrowest CSA, intra-bag pressure, and intra-bag volume were measured for at least 30 s on each FLIP bag distension. The CSA was measured at 16 sites along the axial length of the saline filled bag. The DI (mm^2^/mmHg) was calculated as the median value of the CSA divided by the corresponding intra-bag pressure. The FLIP measurements were analyzed in real time by two expert endoscopists who had an experience of 31 cases of FLIP procedure. After POEM, the FLIP measurement was intraoperatively performed again using the same method. We considered the final DI at 4.0 mm^2^/mmHg ≤ at a balloon distension of 40 or 50-mL an adequate myotomy.

### Assessment of clinical outcomes after POEM

The clinical outcomes were assessed at 3 months and 12 months after POEM. Post-POEM evaluation at 3 months included symptom, HRM, TBE, and esophagogastroduodenoscopy. The symptom improvement was analyzed using Eckardt score, which takes into account weight loss, dysphagia, chest pain, and regurgitation [12]. An Eckardt score after treatment of 3 ≤ was considered a poor response. Follow-up at 12 months after POEM included Eckardt score through outpatient clinic visit and telephone questionnaire.

HRM (Sierra Scientific Instruments, Inc., Los Angeles, CA, USA) was performed for diagnostic purposes before and 3 months after POEM to evaluate the improvement in the integrated relaxation pressure (IRP). Normal LES relaxation on HRM was defined as an IRP ≤ 15 mmHg. On TBE, the column height 5 min after swallowing was evaluated in all patients before and 3 months after POEM. A decrease of 50% < was regarded as significant improvement. Reflux esophagitis was evaluated by esophagogastroduodenoscopy at 3 months after POEM. The grade of reflux esophagitis was evaluated using the Los Angeles classification [13]. Also, all patients prescribed proton pump inhibitor to prevent post-procedure reflux esophagitis after POEM.

The primary outcome of this study was the association of intraoperative final FLIP measurements and clinical response assessed by Eckardt score at 3 months. The secondary outcomes were the correlation of the final values of FLIP with the results of HRM and TBE. We also analyzed the FLIP values according to post-POEM reflux esophagitis.

### Systematic literature review

This systematic review was conducted in accordance with the Preferred Reporting Items for Systematic Reviews and Meta-analyses (PRISMA) guidelines [14]. We systematically searched MEDLINE, the Cochrane Central Register of Controlled Trials, and the Cochrane Database of Systematic Reviews (CDSR) through March 2019. We included all studies that reported on patients with achalasia who underwent intraoperative FLIP measurement and provided clinical outcomes after at least a few weeks of follow-up. Given the diversity of methods used to assess therapeutic outcomes, the studies evaluated clinical outcomes using Eckardt score were included. The therapeutic modalities employed included PBD, LHM, and POEM. “Post-treatment FLIP measurement” was defined as FLIP intraoperatively measured either immediately after the procedure. Selection criteria for inclusion of relevant publications were observational studies, case series, or randomized clinical trials. Searches were limited to studies of human and written by English. We excluded 1) pediatric studies, 2) works employing other therapeutic modalities, 3) studies lacking FLIP data obtained immediately after the procedure, and/or 4) works that did not report Eckardt scores. The search terms were: (“FLIP” OR “Functional lumen imaging probe” OR “impedance planimetry” OR “esophagogastric junction distensibility” OR “esophageal distensibility”) AND (“achalasia”). References were independently reviewed in terms of eligibility by two authors (H.J.G and S.H.K). Disagreements between reviewers were resolved by mutual consensus with corresponding author. The flow chart of the study selection process was demonstrated in S1 Fig. The following information was extracted from each study by the reviewers, author, year of publication, journal, study design, country, number of patients, follow up periods, therapeutic modality, used FLIP probe, used FLIP balloon volume, post-treatment Eckardt score, FLIP measurements. Assessed outcomes was the correlation between the post-treatment FLIP measurements (CSA, DI) and the post-therapeutic clinical outcome using Eckardt score. Quality assessment scale for randomized control trials or cross-sectional studies were unsuitable for the studies included in this review, we assessed the quality of the included studies using modified quantitative quality assessment criteria by Nagpal et al [15, 16]. (S1 Table)

### Statistical analysis

All recorded data measured by EndoFLIP were saved as text files in MATLAB (MathWorks Inc., Natick, MA, USA). Continuous data are expressed as means ± standard deviation (SD) or as the median [interquartile range (IQR)]. Paired data were analyzed using the Wilcoxon signed rank test or paired t-test. Unpaired data were compared using the Mann–Whitney U-test. The values of CSA and DI were present with median and IQR, because the measurements were not normally distributed. The values of CSA and DI were compared using the Mann-Whitney U-test. A P value < 0.05 was considered to indicate statistical significance. The data were analyzed using SPSS version 20.0 (IBM Corp., Armonk, NY, USA).

## Results

### Baseline characteristics of the patients with achalasia and the results of POEM

A total of 23 patients (males, 11), diagnosed with achalasia and of mean age 46.7 ± 15.1 (mean ± SD) years, underwent POEM and intraoperative FLIP measurement. The clinical characteristics and pre-POEM diagnostic parameters are summarized in Table 1. POEM was successfully performed in all patients, without serious adverse events. The clinical success rate 3 months after POEM was 91.3%. Two patients exhibited poor clinical responses (Eckardt scores = 3). The two poor responders, aged 34 and 37 years old, complained of 36 and 12 months of symptom, respectively. They did not undergo prior therapeutic procedure. Also, in initial examination, they did not show sigmoid esophagus. In HRM, both of them showed type II achalasia.

**Table 1:** The patients’ characteristics and clinical outcomes of peroral endoscopic myotomy.

Variables	N = 23
Age, mean ± SD, year	46.7 ± 15.1
Sex, male, n (%)	11 (47.8)
Achalasia subtype	
Type I	6
Type II	14
Type III	3
Previous treatment of achalasia, n	
Pneumatic dilatation	4
Botulinum toxin injection	1
Symptom duration, mean ± SD, month	26.6 ± 21.5
Initial Eckardt score, median [IQR]	6 [4]
Initial IRP on HRM, mean ± SD, mmHg	36.9 ± 13.8
Initial barium column height on TBE, mean ± SD, cm	16.0 ± 10.4
Initial CSA on FLIP at 50-mL balloon, median [IQR], mm^2^	45 [82]
Initial DI on FLIP at 50-mL balloon, median [IQR], mm^2^/mmHg	1.4 [2.4]
POEM procedure time, mean ± SD, minutes	119.2 ± 30.2
Technical success rate of POEM, n (%)	23 (100)
Clinical success rate (Eckardt score < 3), 3 months after POEM, n (%)	21 (91.3)
Follow up duration, median (IQR), month	17.5 [19.0]

SD, standard deviation; IRP, integrated relaxation pressure; HRM, high resolution manometry; TBE, timed barium esophagogram; IQR, interquartile range; CSA, cross -sectional area; DI, distensibility index; POEM, peroral endoscopic myotomy

### The intraoperative FLIP measurements and the clinical outcome at 3 months after POEM

All 23 patients underwent intraoperative impedance planimetry using FLIP before and immediately after POEM. However, in three patients, postoperative FLIP measurements could not be conducted because the intra-bag pressure was < 5 mmHg. Therefore, postoperative esophageal distensibility was measured in 20 patients. The final FLIP values according to clinical response and post-POEM reflux esophagitis were described in Table 2. POEM resulted in improved CSA (from 45 [82] to 148 [162] mm^2^ at 50-mL balloon distension, median [IQR]), and the EGJ-DI (from 1.4 [2.4] to 5.0 [3.7] mm^2^/mmHg at 50-mL balloon distension, median [IQR]). The final CSA and DI did not differ significantly between good and poor responders 3 months after POEM. Post-POEM reflux esophagitis (diagnosed endoscopically) developed in 10 patients. The final CSA and DI did not differ significantly in patients with and without reflux esophagitis.

**Table 2:** Intraoperative final FLIP measurements according to clinical response and post-POEM reflux esophagitis at 3 months after POEM.

	Clinical outcome at 3 months		P value^a^	Post-POEM GERD		P value^a^
	Good response (Eckard score < 3, n = 18)	Poor response (Eckard score 3 ≤, n = 2)		Reflux esophagitis (n = 10)	No reflux esophagitis (n = 10)	
Post-POEM CSA (50-mL), median [IQR], mm^2^	147.0 [147.5]	215 [167.0–263.0]	0.316	156.0 [151.0]	151.0 [177.75]	0.853
Post-POEM DI (50-mL), median [IQR], mm^2^/mmHg	5.01 [4.52]	4.91 [3.63–6.20]	0.853	6.20 [5.15]	4.23 [1.79]	0.075

CSA, cross-sectional area; DI, distensibility index; IQR, Interquartile range

The CSA and DI were measured at 50-mL balloon dilatation.

^a^The data in both groups were compared using the Mann-Whitney U-test.

**Fig 1:**
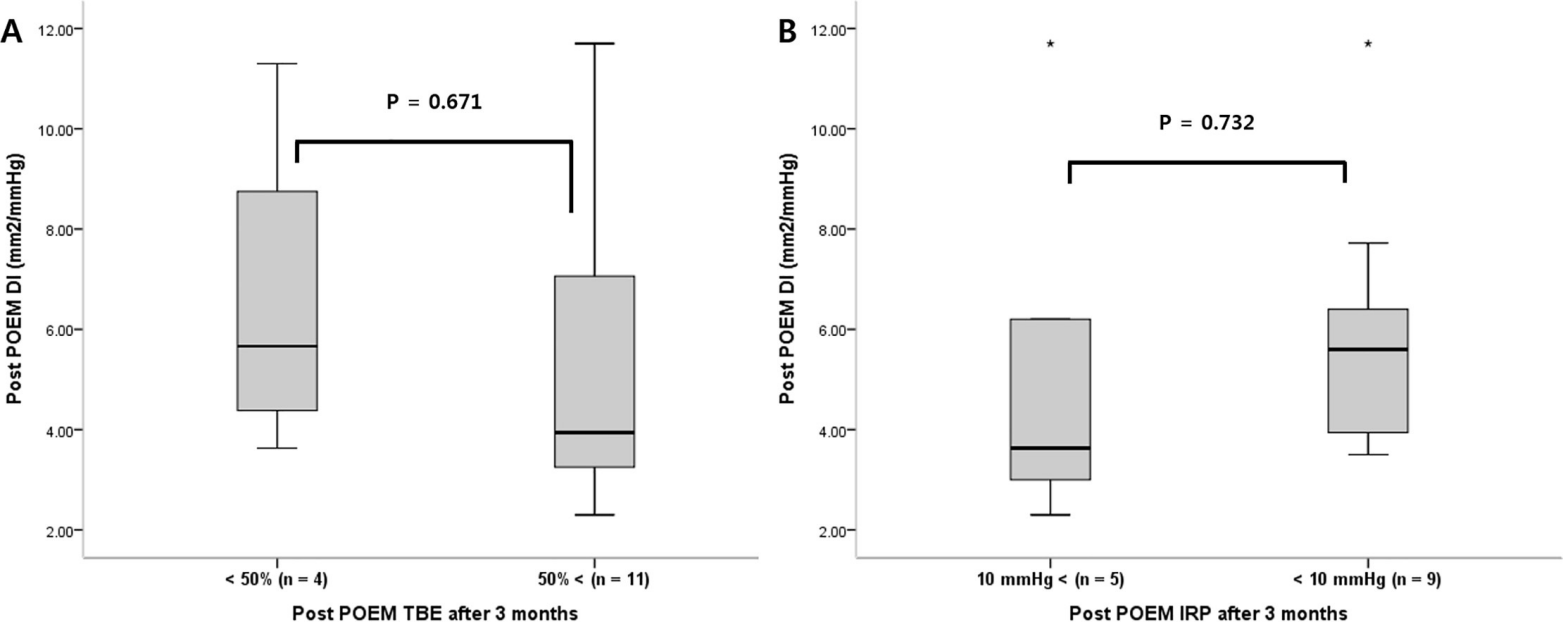
Intraoperative post-POEM distensibility index (DI) based on TBE (timed barium esophagogram) and HRM (high resolution manometry). (A) Intraoperative post-POEM DI and TBE at 3 months after POEM. The results of TBE was classified into two groups, post-procedural reduction in esophageal stasis 50% < or not. (B) Intraoperative post POEM DI and IRP at 3 months after POEM. Statistically significant differences are described by P value.

TBE and HRM at 3 months were performed in 15 and 14 patients, because other patients refused to undergo these diagnostic procedures. The final EGD-DIs and the TBE and HRM data are shown in Fig 1. The TBE results were divided into two classes. A reduction of 50% < in the barium column height was considered to predict a good clinical outcome. Also, an IRP < 10 mmHg on follow-up HRM was considered to reflect a good response. The final EGJ-DI at a 50-mL fill volume did not differ significantly between good and poor responders identified by either TBE or HRM (Fig 1).

### The patients with poor clinical response at 3 months and 12 months after POEM

For the two patients exhibiting poor clinical response through Eckardt score at 3 months, we performed follow-up FLIP assessment 3 months after POEM. FLIP evaluation was performed via endoscopy with the patients under conscious sedation. For one patient (aged 34 years), the final DI at a 50-mL fill volume was 3.63 mm^2^/mmHg and the final CSA 167 mm^2^ during POEM. The follow-up DI at a 50-mL fill volume was 3.3 mm^2^/mmHg and the CSA 142 mm^2^ after 3 months. For the other patient (aged 37 years), the figures were 6.2 mm^2^/mmHg and 263 mm^2^; and 4.1 mm^2^/mmHg and 197 mm^2^. Both patients were recommended an additional PBD. At 12 months after POEM, a patient with Eckardt score of 0 at 3 months complained of dysphagia and regurgitation (Eckardt score = 3). On TBE, the column height at 5 minutes was 8.6 cm. During POEM, the pre- and post-POEM CSA in the patient were 41 mm^2^ and 119 mm^2^ at 50-mL fill volume and DI of 0.7 mm^2^/mmHg and 2.86 mm^2^/mmHg, respectively. The patients were recommended an additional PBD.

## Discussion

We evaluated the role of intraoperative FLIP measurement during POEM in terms of predicting clinical outcomes at 3 months after POEM. POEM significantly improved both the CSA and DI but the final FLIP parameters did not differ significantly between good and poor responders. Evaluation of EGJ physiology after POEM is important for predicting the long-term clinical outcome and in deciding whether re-treatment is needed. Previously, the Eckardt score, LES pressure on HRM, and esophageal stasis on TBE were used to assess the therapeutic outcome and predict the long-term clinical outcome. Symptom assessment using the Eckardt score alone is not adequate because, while patients might adapt to the impaired esophageal function, esophageal deformation may progress during adaptation, either without symptoms or with progressively worsening symptoms. HRM is a gold standard diagnostic modality for achalasia. An IRP of 15 mmHg < on HRM is a necessary parameter in the diagnosis of achalasia because it represents impaired LES relaxation. Also, a previous study showed that an improvement of IRP < 10 mmHg correlated with symptomatic outcomes after POEM [17]. However, according to the recent data, esophageal emptying is impaired in some patients with achalasia but whose IRP is in the normal range on HRM [4, 18]. A post-procedural reduction in esophageal stasis 50% <, as determined on TBE, is considered to indicate long-term symptom remission [19]. The authors reported that remission was not maintained for more than 1 year in patients with a post-procedural reduction in esophageal stasis < 50%, even though neither of those patients complained of symptoms at the time of TBE. However, HRM and TBE are usually performed a few weeks to months after a procedure.

A novel device, termed the FLIP, measures the CSA and the corresponding distensive pressure of the EGJ, which is the therapeutic focus when treating achalasia [20]. FLIP has been used to evaluate therapeutic outcomes after achalasia treatment. Two studies found that EGJ distensibility was significantly higher in patients who maintain good responses than in those maintaining poor responses and treatment naiive patients [2, 3]. In their data, the therapeutic methods and follow-up durations were different in each patient. Verlaan et al. reported that POEM improved EGJ distensiblity, as revealed by FLIP evaluation 3 months after treatment [21]. One study measured post-treatment FLIP at a month after POEM. In this study, post POEM DI < 7 mm^2^/mmHg represented 14.10 of the odds ratio (OR) for poor clinical response [22]. However, in order to be used not only for objective assessment after treatment, but also for predicting the risk of re-treatment, post-treatment FLIP parameters must be compared between good and poor responders.

In this study, we found that the intraoperative final FLIP measurement did not significantly differ between the patients showed good and poor response assessed by Eckardt score, HRM, and TBE at 3 months after POEM. Also, in terms of the post-POEM reflux esophagitis, there was no significant difference in the final CSA and DI. Our systematic literature review revealed similar results to our data. We found a total of five studies including 222 achalasia patients, assessed intraoperative FLIP measurements comparing clinical outcome using Eckardt score (Table 3) [9, 23–26]. Among them, clinical follow-up was ultimately achieved in 192 patients (Table 4). These studies performed intaoperative FLIP during POEM, PD, and LHM. In the three studies, the final EGJ diameter and DI were higher in good responders, but it was not significantly different [23, 25, 26]. The other two studies did not show the final FLIP values between good and poor responders directly [9, 24]. However, in the study of Smeets et al., they found no difference in the final DI during PD between good and poor responders at 1-year follow-up [24]. In another study of Teitelbaum et al., the increase in DI was significantly larger in lower post-LHM Eckardt score group (P < 0.05) and no significance in post-POEM group [9].

**Table 3:** Characteristics of included studies.

Author	Year	Country	Study design	Number of patients	Follow up periods (monmon((months)	Therapeutic modality	FLIP probe	FLIP balloon volume (mL)	Sedation
Familiari et al. [23]	2014	Italy	Prospective	23 (21)^a^	5 months	POEM	EF-325N	30, 40	General anesthesia
Smeets et al. [24]	2015	Netherlands	Prospective	26 (22)^a^	12 months	PD	EF-325N	30, 40, 50	Conscious Sedation
Teitelbaum et al. [9]	2015	USA	Prospective	56 (32)^a^	12–11 months	LHM (20/11) POEM (36/21)	EF-325N	40	General anesthesia
Ngamruengphong et al. [25]	2016	USA, Germany	Retrospective	63	3–6 months	POEM	EF-325N	30, 40	General anesthesia
Wu et al. [26]	2018	Australia	Prospective	54	2 weeks	PD	EF-325N	40	Conscious Sedationn

FLIP, functional lumen imaging probe; POEM, peroral endoscopic myotomy; PD, pneumatic dilatation; LHM, laparoscopic heller myotomy

^a^(), The numbers in parentheses indicate the number of patients ultimately followed up for clinical outcome among total number of patients.

**Table 4:** Summary of intraoperative FLIP measurements in patients treated for achalasia.

Authors	Definition of good response	Measurements of intraoperative final FLIP	Final FLIP values in good responders	Final FLIP values in poor responders	P value
Familiari, et al.	Eckardt score < 1	EGJ diameter, mm^2^ (mean ± SD)	11.689 ± 1.822	11.067 ± 1.707	P = 0.432
Smeets et al.	Eckardt score < 4	EGJ DI, mm^2^/mmHg	NA*	NA	NA
Teitelbaum, et al.	Eckardt score ≤ 1	Δ EGJ DI, mm^2^/mmHg	NA	NA	NA
Ngamruengphong, et al.	Eckardt score < 3	EGJ DI, mm^2^/mmHg (median, range)	5.95 (4.55–8.90)	2.95 (1.00–9.90)	P = 0.26
Wu et al.	Eckardt score ≤ 3	EGJ DI, mm^2^/mmHg (mean)	6.5	5.8	P = 0.51

FLIP, functional lumen imaging probe; EGJ, esophagogastric junction; SD, standard deviation; DI, distensibility index; NA, not available

In our data, two patients revealed poor response at 3 months (Eckardt score = 3) and a patient at 12 months (Eckardt score = 3). We performed follow-up FLIP measurements in the two patients exhibiting poor clinical outcomes at 3 months after POEM. The follow-up EGJ-DI was reduced compared to the intraoperative final DI. In a patient of the two patients, 37 year-old female, mucosal flap injury occurred during POEM. Thus, although the EGJ DI immediately after POEM increased, fibrosis in the wound healing process for 3 months after the procedure would have caused EGJ DI reduction in follow-up FLIP. It is conceivable that EGJ DI may fall during wound-healing after operation because EGJ distensibility is affected by the compliance of surrounding tissues. Therefore, it may be that, in some patients, the intraoperative FLIP does not reflect structural changes caused by postoperative wound-healing. However, more data on intraoperative and follow-up FLIP measurements are required.

Despite the current ambiguity of any role for intraoperative FLIP, the technique can be used during operation. As FLIP can be applied during a procedure or an operation, unlike HRM or TBE, FLIP has been considered to measure EGJ distensibility during LHM, PBD, or POEM. One study comparing the use of intraoperative FLIP during LHM with endoscopy-assisted LHM found that employment of FLIP during operation reduced the duration of myotomy without compromising clinical outcomes [27]. In another studies, EGJ distensibility changed at each operative step of LHM and POEM, thus after myotomy, dissection, and further myotomy [7, 8]. Therefore, it is conceivable that EGJ distensibility measured during an operation or procedure may indicate the extent of myotomy adequate to afford symptom relief. In this study, we targeted final EGJ DI at 4 mm^2^/mmHg ≤ at 40 or 50-mL balloon distension to improve symptom and to prevent post-POEM reflux esophagitis according to the previous published data [3, 24, 28]. A patient did not satisfy the target DI (3.4 mm^2^/mmHg at 40-mL and 2.3 mm^2^/mmHg at 50-mL), but the scope resistance after myotomy was reduced. Therefore, we did not perform additional myotomy after FLIP measurement in all patients.

To use intraoperative FLIP for tailored myotomy, target values ensuring symptom improvement and the absence of post-treatment reflux esophagitis are required. Before choosing such values, it is essential to show that intraoperative FLIP predicts the postoperative clinical outcome. Previous efforts to define associations between intraoperative or intraprocedural FLIP measurements, and short- and long-term clinical outcomes, have been inconclusive. Teitelbaum et al. suggested that the ideal final intraoperative DI range was 4.5–8.5 mm^2^/mmHg for both LHM and POEM. Patients within this range exhibited adequate clinical outcomes (Eckardt scores ≤1 and GERD scores ≤ 7) [9]. Previously suggested target values must be verified in a larger group followed-up long-term. Additionally, the protocol must be standardized. Currently, DI is commonly calculated to assess treatment effectiveness. However, as reported in a recent study on PBD, the delta DI value measured during the procedure can be considered [26].

This study has several limitations. First, we included only a small number of patients, and only two responded poorly. Also, the follow-up time was short. Therefore, it was difficult to conclude that intraoperative FLIP played any useful role. Second, although we planned to assess the results of TBE and HRM after POEM as well as Eckardt score, we couldn’t perform the diagnostic procedures in all patients. However, we included these patients in this study, because the primary outcome was the association between final FLIP and clinical outcome assessed by Eckardt score. Third, previous studies in the systematic review showed heterogeneity in procedure protocol, target parameters, and outcome evaluation. Such heterogeneity renders it difficult to compare the results from a statistical viewpoint. However, as published data on intraoperative FLIP measurements are scarce, further studies with larger groups and evaluation of long-term clinical outcomes are required.

## Conclusions

In conclusion, FLIP can be used to assess changes in EGJ distensibility during POEM. However, we found no significant correlation between the final intraoperative FLIP parameters and the clinical response, in agreement with literature data. Thus, although intraoperative FLIP measurement may usefully reflect the adequacy of myotomy during POEM, a further study with more patients is necessary to explore whether FLIP can predict short- and long-term clinical responses.

## Supporting information

S1 Checklist PRISMA checklist. (DOC)

S1 Fig Flow chart for the systematic review. (TIF)

S1 Table Quality assessment criteria for the included studies. (DOCX)

S1 File Spreadsheet of raw data. NA, not available. ^a^ In three patients, postoperative FLIP measurements could not be conducted because the intra-bag pressure was < 5 mmHg. ^b^ In four patients, the initial IRP on HRM could not measure because the probe could not pass through the EGJ due to the high pressure. ^c^ TBE and HRM were performed in 15 and 14 patients, because other patients refused to undergo these diagnostic procedures. ^d^ In two patients, Eckardt score could not evaluated due to follow-up loss. ^e^ In eight patients, the FLIP measurements was only performed in 50-mL balloon distension.
(XLSX)
