# Supplementary figures and images for: Intraoperative use of a functional lumen imaging probe during peroral endoscopic myotomy in patients with achalasia: A single-institute experience and systematic review
Source: PLoS One. 2020 Jun 9;15(6):e0234295. doi: 10.1371/journal.pone.0234295 (PMC7282640; doi:10.1371/journal.pone.0234295)

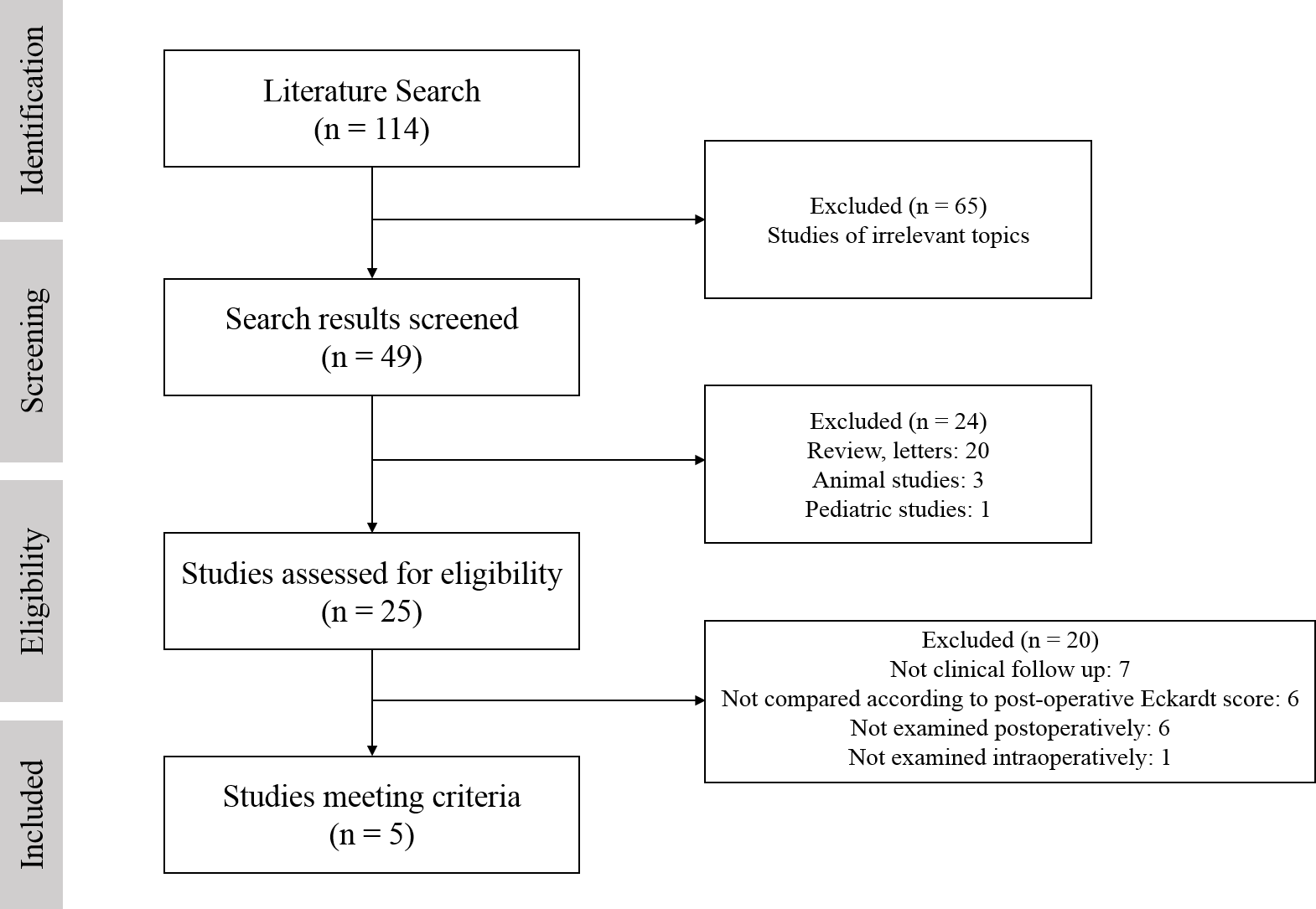

Supplement: S1 Fig — (TIF) [file pone.0234295.s002.tif]
