# Supplementary material for: Intraoperative use of a functional lumen imaging probe during peroral endoscopic myotomy in patients with achalasia: A single-institute experience and systematic review
Source: PLoS One. 2020 Jun 9;15(6):e0234295. doi: 10.1371/journal.pone.0234295 (PMC7282640; doi:10.1371/journal.pone.0234295)
Supplement: S1 Table — (DOCX) [file pone.0234295.s003.docx]

**Supplementary table 1.** Quality assessment criteria for the included studies

| Criteria | 0 | 1 | 2 |
| --- | --- | --- | --- |
| Are the aims of the study clearly stated? | Aims not stated | Some aims stated clearly/all aims stated unclearly/not in abstract | All aims stated clearly & included in abstract |
| Are the inclusion and exclusion criteria for participants included? | No mention of participant criteria | Only either inclusion or exclusion criteria included | Both inclusion & exclusion criteria included |
| Are participant demographic characteristics included? | No reference to participant characteristics | Some reference to participant characteristics | Detailed references to participant characteristics |
| Are the procedures described in detail? | No mention of procedures | Brief mention of procedure | Explanation of whole procedure |
| Description of how themes identified? | No description of analysis | Named theory/brief description | Explanation of how themes/analysis conducted |
| Was ethical approval obtained and acknowledged? | No reference to ethical approval | Ethical approval from unnamed body | Ethical approval gained from a named body |
| Were power calculations performed? | No mention of power calculations performed | Power calculation attempted | Study numbers match power calculation |
| Are statistics appropriate? | No statistics/inappropriate statistics | Appropriate statistics but no justification given | Appropriate statistics used with justification |
| Are p values included? | No p values included | p values included, but not all exact/not all p values included | All p values included with exact values |
| Are limitations of the study acknowledged? | No limitations acknowledged | Some but not all limitations acknowledged/acknowledgement of limitations unclear | Limitations acknowledged |
